# Treatment outcomes and prognostic factors of esthesioneuroblastoma—a retrospective study from South India

**DOI:** 10.3332/ecancer.2023.1584

**Published:** 2023-07-27

**Authors:** Lekha Madhavan Nair, John Mohan Mathew, Malu Rafi, Kainickal Cessal Thommachan, Jagathnath Krishna KM, Bipin T Varghese, Rejnish Ravikumar

**Affiliations:** 1 Department of Radiation Oncology, Regional Cancer Centre, Thiruvananthapuram 695011, India; 2 Department of Epidemiology and Biostatistics, Regional Cancer Centre, Thiruvananthapuram 695011, India; 3 Department of Surgical Oncology, Regional Cancer Centre, Thiruvananthapuram 695011, India

**Keywords:** esthesioneuroblastoma, survival, surgery, radiotherapy

## Abstract

Esthesioneuroblastoma (ENB) or olfactory neuroblastoma is a rare malignant neoplasm arising from the neural crest cells of the olfactory epithelium. The optimum treatment for this rare disease is still unclear. Most of the available literature on this rare head and neck tumour is limited to small retrospective series and single institutional reports. We conducted a retrospective study to investigate the clinical profile, treatment outcomes and prognostic factors of patients with ENB treated at a tertiary cancer centre in south India. Patients with a histopathological diagnosis of ENB treated from 2000 to 2019 were included. Patient demographics, tumour characteristics, stage, treatment details and outcome data were identified from medical records. Overall survival (OS) and disease-free survival (DFS) were estimated using the Kaplan–Meier method and the log-rank test was used for comparison. The prognostic factors were identified using Cox regression analysis. Forty-two patients underwent treatment for ENB from 2000 to 2019. Twenty-six patients underwent surgery. Twelve patients received radical radiotherapy (RT) while 24 patients underwent adjuvant radiation. After a median follow-up of 71 months, the estimated OS and DFS at 4 years were 64.4% and 54%, respectively. The estimated 4-year OS for modified Kadish A, B, C and D stages was 75.0%, 90.9%, 56.4% and 0%, respectively. Modified Kadish stage, nodal involvement, orbital invasion, intracranial extension, surgery, RT treatment and use of chemotherapy were significant predictors of OS and DFS in univariate Cox regression analysis. Orbital invasion and RT treatment were significant predictors of DFS in the multivariate analysis as well. However, only RT treatment came out to be a significant predictor for OS in multivariate Cox regression analysis. Surgery is the mainstay of treatment. Adjuvant RT may improve local control and survival in advanced cases. Advanced modified Kadish stage, lymph node involvement and orbital invasion are associated with poor outcomes.

## Introduction

Esthesioneuroblastoma (ENB) or olfactory neuroblastoma is a rare malignant neoplasm arising from the neural crest cells of the olfactory epithelium within the nasal cavity [1, 2]. It accounts for 3% of all nasal cavity tumours [3]. The aetiology of this rare round cell tumour is unclear, but it is postulated that viral, genetic and environmental factors play a role in its development [4]. ENB has a varying clinical course, ranging from indolent disease to a highly aggressive neoplasm with local infiltration and distant metastasis.

The optimum treatment for this rare disease is still unclear, with surgery being the mainstay in managing these tumours. The complex anatomical location and the difficulty in achieving a clear surgical margin due to the infiltrative nature of the lesion often pose challenges in treatment [5]. Radiotherapy (RT) is an integral part of the multidisciplinary management of this disease and several retrospective studies have reported improvement in local control with adjuvant RT [3, 6]. The role of chemotherapy is not completely understood in the multimodal setting, relating to tumour stage and pathology. Even though there are reports suggesting good response rates with neoadjuvant chemotherapy in locoregionally advanced disease [7], there are retrospective series that point towards poor outcomes with the use of chemotherapy [1, 8].

Most of the available literature on this rare head and neck tumour is limited to small retrospective series and single institution reports and only limited information is available about the prognostic factors and treatment outcomes. We aimed to investigate the clinical profile, treatment outcomes and prognostic factors of patients with ENB treated at a tertiary cancer centre in south India.

## Materials and methods

This was a retrospective study conducted at a tertiary cancer centre in India. Patients with a histopathological diagnosis of ENB treated from 2000 to 2019 were included in the study. Patients aged less than 13 years were excluded from the study. Patient demographics, tumour characteristics, stage, treatment details and outcome data were identified from medical records. Modified Kadish staging and Dulguerov staging were done for all patients. The information on Hyams grade was not available for all patients and hence its prognostic significance was not assessed. Cross-sectional imaging of the head and neck (contrast-enhanced magnetic resonance imaging or contrast-enhanced computed tomography) was used for staging. Surgery was done for all resectable tumours. Patients with Kadish stages B, C and D received adjuvant RT. Induction chemotherapy was given for unresectable tumours and concurrent cisplatin was given as a part of radical chemoradiation. The common induction regimens were cisplatin-etoposide and vincristine, adriamycin and cyclophosphamide (VAC).

Patients were followed up with clinical examinations every 3 months during the first 2 years and every 6 months thereafter. Cross-sectional imaging was done as and when indicated. Follow-up was updated using clinical and telephonic information. The study was approved by the Institutional Review Board (SRC no: 8/2022/03).

### Statistical analysis

The categorical variables were presented using values and percentages. Continuous variables were summarised using mean, median and standard deviation. Overall survival (OS) and disease-free survival (DFS) were estimated using Kaplan–Meier method and the log-rank test was used for comparison. The prognostic factors were identified using Cox regression analysis. OS was calculated from the date of diagnosis to the date of death or last follow-up. DFS was computed from the date of diagnosis to the date of relapse, progression of disease or death. The Cox regression model was used to identify the statistical prognostic factors. Statistical analysis was done using Statistical Package for the Social Sciences software version 11.0.

## Results

Forty-two patients underwent treatment for ENB from 2000 to 2019. There were 24 males (57.1%) and 18 females (42.9%). The median age was 47 years (13–78). Table 1 shows the patient and tumour characteristics. No patient had metastatic disease at presentation. Twenty-six (61.9%) patients underwent surgery. Endoscopic surgery was done on 16 patients and 10 patients underwent open surgery. Sixteen patients underwent a biopsy only. Out of the 20 patients with intracranial extension, 7 underwent surgery. Open craniofacial resection was done for four patients, whereas endoscopic surgery was done for three patients. Six patients underwent adjuvant RT after surgery. Out of the 12 patients with orbital invasion, two underwent surgery, which amounted to orbital exenteration. Thirty-six patients (85.7%) underwent RT. Twelve patients received radical RT while 24 patients underwent RT as adjuvant treatment. Seventeen patients received intensity-modulated RT, seven patients received conformal RT and others received conventional treatment. RT doses ranged from 50 to 70 Gy as conventional fractionation. Chemotherapy was given to 18 patients (42.9%), as neo-adjuvant for 15 patients and concurrent (cisplatin) for 2 patients. One patient received adjuvant chemotherapy with cisplatin and 5-fluorouracil. Cisplatin-etoposide was the most common neo-adjuvant regimen used, followed by the VAC regimen. Two patients underwent surgery plus adjuvant RT and eight patients received radical RT after neoadjuvant chemotherapy. Five patients developed disease progression after neoadjuvant chemotherapy (before the local treatment) and they received palliative treatment. Table 2 shows the details of treatment received according to the modified Kadish stage.

### Treatment

Among the 12 patients who underwent RT alone, 8 attained a complete response, 1 attained a partial response, 2 had stable disease and 1 developed disease progression. Out of the 26 patients who underwent surgery as their treatment (surgery + RT or surgery alone), 4 developed local recurrences, 3 developed regional recurrences and 1 developed distant relapse. Five out of 16 patients had disease progression after neoadjuvant chemotherapy. Distant metastases developed in 3 out of the total 42 patients, with the sites of metastases being the liver and lungs.

### Survival

**Figure 1.**
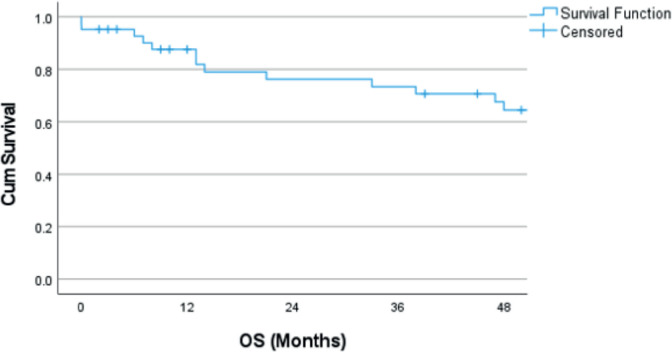
Kaplan–Meier curve showing OS probability. OS: Overall survival.

**Figure 2.**
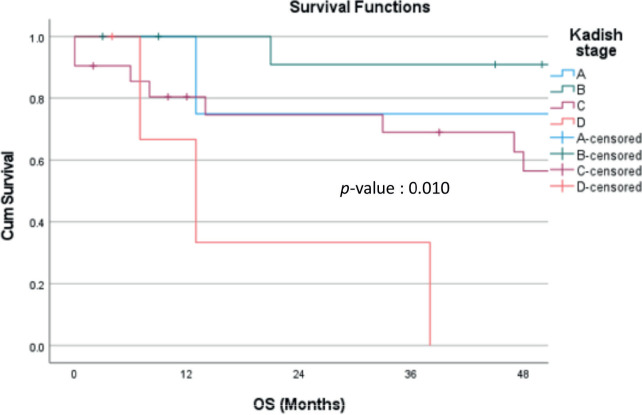
OS probability according to modified Kadish stages (p-value 0.010). OS: Overall survival.

**Figure 3.**
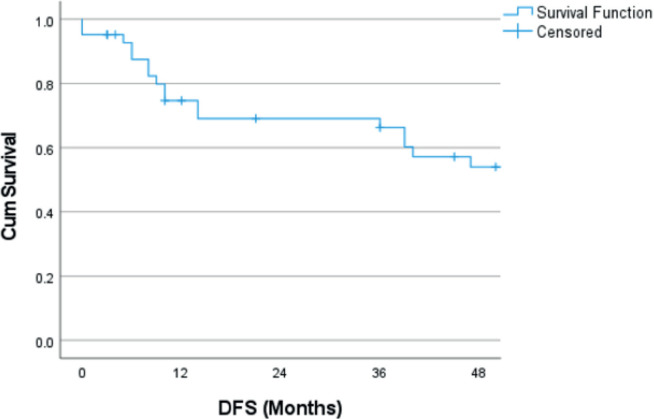
Kaplan–Meier curve showing DFS probability. DFS: Disease free survival.

The median follow-up was 71 months (2–230 months). The estimated 4-year OS was 64.4% (Figure 1). The estimated 4-year OS for modified Kadish A, B, C and D stages is 75.0%, 90.9%, 56.4% and 0%, respectively (Figure 2). Age is evaluated as a continuous variable in the present study and a cutoff value of 40 years was obtained using Receiver Operating Characteristic (ROC) curve analysis. No significant difference in OS or DFS was noticed in patients aged <40 years versus ≥40 years. A significant difference in OS was noticed for the modified Kadish stage, T stage, nodal stage, presence of orbital invasion, presence of intracranial extension, surgical treatment, use of adjuvant or radical RT and chemotherapy treatment (Table 3). The estimated 4-year DFS was 54% (Figure 3). There was a significant difference in DFS probability according to the modified Kadish stage, presence of orbital invasion, presence of intracranial extension, surgical treatment and use of RT and chemotherapy (Table 4). Advanced modified Kadish stage [hazard ratio (HR) 9.419, 95% confidence interval (CI) 0.917–96.785, *p*-0.036], presence of nodal involvement (HR 5.820, 95% CI 1.512–22.406, *p*-0.010), presence of orbital invasion (HR 4.131, 95% CI 1.476–11.567, *p*-0.007), presence of intracranial extension (HR 3.340, 95% CI 1.135–9.826, *p*-0.029) and use of chemotherapy (HR 4.184, 95% CI 1.419–12.337, *p*-0.009) were associated with decreased survival in univariate Cox regression analysis (Table 5). Patients who underwent surgery (HR 0.173, 95% CI 0.059–0.512, *p*-0.002) and those who received RT as adjuvant or radical treatment (HR 0.220, 95% CI 0.067–0.729, *p*-0.013) were significantly associated with better OS. The use of radical or adjuvant RT was associated with better survival in multivariate analysis as well (HR 0.105, 95% CI 0.025–0.433,* p*-0.002). Univariate Cox regression analysis for DFS (Table 5) showed significant association with nodal involvement (HR 3.892, 95% CI 1.065–14.227, *p*-0.040), presence of orbital invasion (HR 3.175, 95% CI 1.267–7.960, *p*-0.014), presence of intracranial extension (HR 2.909, 95% CI 1.134–7.463, *p*-0.026) and the use of chemotherapy (HR 3.326, 95% CI 1.304–8.483, *p*-0.012). Patients who underwent surgical treatment (HR 0.293, 95% CI 0.117–0.730, *p*-0.008), and those who received adjuvant or radical RT (HR 0.153, 95% CI 0.053–0.440, *p*-0.001) were associated with significantly better DFS (Table 4). The presence of orbital invasion (HR 3.051, 95% CI 1.197–7.773, *p*-0.019) and use of RT treatment (HR 0.158, 95% CI 0.054–0.463, *p*-0.001) were significant predictors of DFS in the multivariate analysis also.

## Discussion

ENB is a malignant neoplasm of the nasal cavity with varying understanding of this rare tumour in published literature. We are reporting on the treatment outcomes of 42 patients treated over a span of 20 years at a tertiary cancer centre in South India.

A bimodal age distribution in the second and sixth decades of life has been reported for ENB [9]. About half of the patients in this study presented in the fourth to sixth decades, with a median age at presentation of 47 years (range 13–78 years). A similar distribution of age was reported in other studies by Yin *et al* [10] and Tural *et al* [11] also. The incidence among males and females was reported to be equal in some studies [11–13] while some others showed a male predilection [14–16]. The male-female ratio in this study was 1.3.

There is no separate American Joint Committee on Cancer/The International Union Against Cancer staging for ENB. Multiple staging systems have been proposed, the most commonly used systems being modified Kadish staging and Dulguerov staging. Most of the patients in this cohort were modified Kadish stage C, similar to other published series [13, 15]. There was a significant difference in DFS and OS according to the modified Kadish stage. The OS for stages A, B, C and D was 75%, 90.9%, 56.4% and 0%, respectively. The 4-year DFS was 75%, 83.3%, 41.3% and 0% for stages A, B, C and D, respectively. There were 4 patients with modified Kadish A stages, of whom 1 developed lung metastasis and died of disease progression, whereas only 2 out of the 13 modified Kadish B patients died of disease. That may be the reason for improved survival in patients with modified Kadish B stage compared to Kadish A. The tumours were staged according to the Dulguerov T staging system as well. But there was no significant difference in OS or DFS according to the T stage. Two large retrospective series reported the Kadish stage as a significant predictor of OS in univariate analysis [3, 17]. A retrospective population-based cohort study by Jethanamest *et al* [18] also showed the modified Kadish stage as a significant factor affecting OS and disease-specific survival. The modified Kadish stage was significantly correlated with OS in the present study as well. Some studies have concluded that the existing staging systems do not accurately predict survival [19, 20]. A new Tumour, Node, Metastasis (TNM) staging system has been proposed by Sun *et al* [21] but has to be validated in future studies.

Orbital invasion, which is staged as C as per the modified Kadish system, is found to be associated with poor prognosis and this was seen in 41.7% of patients in this series. A similar rate of orbital involvement was reported by Song *et al* [17]. The 4-year OS probability for patients with and without orbital invasion was 41.7% and 73.35%, respectively. These figures are lower than the large single institution report by Song *et al* [17]. Only 16% of patients with orbital involvement underwent surgery in the present study. That may be the reason for inferior outcomes compared to the previously published series. Li *et al* [22] categorised patients with orbital invasion into three grades and there was a significant difference in OS and PFS between grades I, II and III lesions in univariate analysis. There was no difference in survival among patients treated with and without surgery and they concluded that radical RT is a viable option for patients with advanced and unresectable tumours extending into the orbit. Surgery followed by adjuvant RT is the recommended treatment for operable tumours. Inoperable lesions may be treated by radical RT.

Olfactory neuroblastoma has a pathway of direct spread to the brain through the cribriform plate. Surgery plays a crucial role in the management of these tumours and adjuvant RT may be beneficial for improved local control. Although 20 patients in this series had an intracranial extension, only 7 underwent surgical treatment, 4 open Cranio-Facial resections and 3 endoscopic surgeries. A systematic review and meta-analysis by Fu *et al* [23] showed that endoscopic approaches have comparable control rates to open craniofacial resection. Patients with intracranial involvement had inferior OS and DFS (45.6% versus 35.1%) and this was reflected in the univariate analysis although no significance was found in the multivariate analysis. The intracranial extension did not influence survival in two previously reported retrospective studies [17, 24].

Cervical lymph nodes are the most frequent sites of regional spread of the disease with level II nodes being the most common [25]. The disease can also spread to levels I, III and retropharyngeal nodes [26, 27]. The incidence of lymph node metastasis was 9.5% which is similar to other published series [3, 12, 14]. Several retrospective studies have reported lymph node metastasis as a significant predictor of outcome [17]. Lymph node involvement was associated with decreased OS and DFS in this study as well. In univariate Cox regression, lymph node involvement was a significant predictor of OS and DFS. Patients underwent neck dissection and nodal irradiation in cases of clinical, radiological or pathological evidence of lymph node involvement. There is no strong evidence for prophylactic neck irradiation [28]. There were three isolated nodal recurrences in this study and all of them were successfully salvaged by surgery and radiation.

There is documented evidence to support the difficulties in achieving negative surgical margins due to the anatomical location and infiltrative nature of this disease and this theoretically opens avenues for the use of adjuvant RT for reducing local recurrence [5]. Even though reports from the SEER (Surveillance, Epidemiology, and End Results) database showed no benefit for adjuvant RT [4], a large population-based database of 931 patients from the University of Utah reported a 47% decrease in risk of death with the addition of RT after surgery [29]. We found that RT, given in either a radical or adjuvant setting, reduced the risk of death and recurrence.

Although chemotherapy is widely used in the neoadjuvant, concurrent and palliative setting, its exact role in the management of these tumours is debatable. The use of neoadjuvant chemo often helps in shrinking the tumour prior to definitive treatment, thereby enabling better resections or safer RT delivery. We found that patients who received chemotherapy had a four times higher risk of death and a 3.3 times higher risk of recurrence compared to patients without chemotherapy treatment in the univariate analysis. This difference was significant in multivariate analysis as well. These findings are in concurrence with the findings of two large population database studies which brought out the detrimental effects of chemotherapy [8, 30]. The poor outcomes for patients who received chemotherapy could be a reflection of the fact that the majority of the patients who were given chemotherapy had either Kadish C or D disease and most had unresectable disease or had difficult RT planning.

Adjuvant or definitive RT is challenging in ENB due to the complex anatomical relations of these tumours which includes vital areas like the optic apparatus, brain stem and the brain. Even though long-term follow-up data is lacking, proton beam therapy and carbon ion therapy are being established as effective and safe treatment alternatives to conventional external beam RT, due to their ability to attain sharp dose falloff beyond the depth of Bragg peak [31–34]. But long-term follow-up data is needed to establish the role of particle beam therapy.

Multiple studies have identified different genetic and cytogenetic alterations in ENB. The most common chromosomal alterations include deletion on chromosome 11 and gain on chromosome 1p, which is associated with poor prognosis [35]. Alterations in p53, EGFR, KIT, RET, APC, FGFR2, PDGFRA, SMAD4 and CTNNB1 genes are also associated with ENB [36–38]. This might provide potential therapeutic pathways by targeting these genetic alterations.

The retrospective nature of the study could have resulted in selection bias. Due to small patient numbers, some statistical evaluations could not be fully done. The study population was sourced over a 19-year period, treatment approaches may have changed during this period.

## Conclusion

The majority of patients with ENB present in advanced stages. Surgery is the mainstay of treatment. Adjuvant RT may improve local control and survival in advanced cases. Advanced modified Kadish stage, lymph node involvement and orbital invasion have prognostic importance.

## List of abbreviations

CI: Confidence interval; DFS: Disease free survival; ECOG: Eastern Co-operative Oncology Group; ENB: Esthesioneuroblastoma; OS: Overall survival; RT: radiotherapy; UICC: The International Union Against Cancer.

## Conflicts of interest

None.

## Funding

None.

## Author contributions

Dr Rejnish Ravikumar, Dr Lekha Madhavan Nair and Dr John Mohan Mathew contributed to study conception and design. Material preparation, data collection and analysis were performed by Dr Lekha M Nair, Dr John Mohan Mathew and Dr Jagathnath Krishna K M. The first draft of the manuscript was written by Dr Lekha M Nair and all authors commented on the previous versions of the manuscript. All authors read and approved the final manuscript.

## Figures and Tables

**Table 1.** Baseline patient characteristics.

	Number	Percentage
Age <40 ≥40	17 25	40.5 59.5
Gender Male Female	24 18	57.1 42.9
ECOG performance status 0 1 2	6 32 4	14.3 76.2 9.5
Modified Kadish stage A B C D	04 13 21 04	9.5 31.0 50.0 9.5
T stage T1 T2 T3 T4	9 9 1 23	21.4 21.4 2.4 54.8
Nodal stage N0 N1	38 4	90.5 9.5
Orbital invasion Yes No	12 30	28.6 71.4
Intracranial extension Yes No	20 22	47.6 52.4
Surgery Surgical treatment No surgical treatment	26 16	61.9 38.1
RT Irradiated Not irradiated	36 06	85.7 14.3
Chemotherapy Yes No	18 24	42.9 57.1

ECOG: Eastern Co-operative Oncology Group

**Table 2.** Treatment received according to the modified Kadish stage.

Modified Kadish stage	Surgery alone	RT alone	Surgery + RT	Chemotherapy (neoadjuvant, concurrent or adjuvant)
A	0	0	4	0
B	1	1	11	0
C	1	8	9	14
D	0	3	0	4

RT: Radiotherapy

**Table 3.** OS probability at 2 and 4 years.

Variables	Group	OS						
		2 years			4 years			
		Survival probability (%)	LCI	UCI	Survival probability (%)	LCI	UCI	*p*-value
Age	<40	73.3	50.76	95.84	73.3	50.76	95.84	0.331
	≥40	78.1	61.048	95.152	59	38.224	79.776	
Gender	Male	73.5	55.272	91.728	63.6	43.216	83.984	0.884
	Female	80.2	60.012	100.388	65.6	40.904	90.296	
Kadish stage	A	75	32.468	117.532	75	32.468	117.532	0.01
	B	90.9	73.848	107.952	90.9	73.848	107.952	
	C	74.7	55.296	94.104	56.4	33.272	79.528	
	D	33.3	3.508	63.092	0	0	0	
T stage	T1	87.5	64.568	110.432	87.5	64.568	110.432	0.052
	T2	87.5	64.568	110.432	87.5	64.568	110.432	
	T3	100	-	-	100	-	-	
	T4	65.9	45.124	86.676	42.7	19.964	65.436	
Nodal stage	N0	79.9	66.572	93.228	70.2	54.52	85.88	0.004
	N1	33.3	3.508	63.092	0	0	0	
Orbital invasion	Yes	55.6	26.396	84.804	41.7	9.556	73.844	0.003
	No	85.2	71.872	98.528	73.3	56.248	90.352	
Intracranial extension	Yes	66.5	44.352	88.648	45.6	20.708	70.492	0.02
	No	85	69.3244	100.688	79.7	61.864	97.536	
Surgery	Yes	91.3	79.736	102.864	82.1	66.224	97.976	<0.001
	No	49.2	15.268	51.332	32.8	6.928	58.672	
RT	Yes	83.9	70.964	96.836	70.1	53.636	86.564	0.006
	No	33.3	15.268	51.332	33.3	15.268	51.332	
Chemotherapy	Yes	55.9	31.008	80.792	41.9	16.812	66.988	0.005
	No	90.5	77.956	103.044	80.4	63.152	97.648	

OS: Overall survival, LCI: Lower limit of confidence interval, UCI: Upper limit of confidence interval

**Table 4.** DFS probability at 2 and 4 years.

Variables	Group	DFS						
		2 years			4 years			
		Survival probability (%)	LCI	UCI	Survival probability (%)	LCI	UCI	*p*-value
Age	<40	67.2	43.484	90.916	47.1	21.816	72.384	0.868
	≥40	70	51.184	88.816	59.6	38.824	80.376	
Gender	Male	69.6	50.784	88.416	50.6	29.628	71.572	0.795
	Female	68.2	45.072	91.328	59.7	34.22	85.18	
Kadish stage	A	75	32.468	117.532	75	32.468	117.532	0.027
	B	83.3	62.132	104.468	83.3	62.132	104.468	
	C	65	44.028	85.972	41.3	18.564	64.036	
	D	33.3	3.508	63.092	0	0	0	
T stage	T1	87.5	64.568	110.432	87.5	64.568	110.432	0.052
	T2	77.8	50.556	105.044	62.2	27.312	97.088	
	T3	100	-	-	100	-	-	
	T4	56.9	35.536	78.264	34.5	12.94	56.06	
Nodal stage	N0	72.1	57.4	86.8	58.8	41.944	75.656	0.08
	N1	33.3	3.508	63.092	0	0	0	
Orbital invasion	Yes	46.3	16.9	75.7	34.7	5.104	64.296	0.009
	No	78.3	62.816	93.784	61.5	42.488	80.512	
Intracranial extension	Yes	55.2	31.876	78.524	35.1	11.58	58.62	0.019
	No	81	64.144	97.856	69.7	49.316	90.084	
Surgery	Yes	79.6	63.528	95.672	69.3	50.092	88.508	0.005
	No	51	24.736	77.264	29.2	5.288	53.112	
RT	Yes	78.5	64.388	92.612	64.2	46.952	81.448	<0.001
	No	16.7	6.508	26.892	0	0	0	
Chemotherapy	Yes	50.8	26.3	75.3	31.7	8.768	54.632	0.007
	No	82.6	67.116	98.084	71.2	51.404	90.996	

OS: Overall survival, LCI: Lower limit of confidence interval, UCI: Upper limit of confidence interval

**Table 5.** Univariate Cox regression for OS and DFS.

Factor	OS				DFS			
	Hazard ratio	CI Lower limit	Upper limit	p-value	Hazard ratio	CI Lower limit	Upper limit	*p*-value
Age (≥40 versus <40)	1.748	0.556	5.5	0.339	0.926	0.370	2.317	0.870
Gender (Female versus male)	1.080	0.384	3.038	0.884	1.130	0.444	2.874	0.797
Kadish B versus A C versus A D versus A	0.690 2.434 9.419	0.062 0.307 0.917	7.626 19.319 96.785	0.036 0.762 0.400 0.059	0.392 1.720 4.510	0.055 0.382 0.703	2.794 7.749 28.921	0.065 0.350 0.480 0.112
T stage T2 versus T1 T3 versus T1 T4 versus T1	2.016 0 7.031	0.182 0 0.910	22.3 0 54.347	0.135 0.568 0.987 0.062	1.832 4.404	0.305 0.992	11.023 19.561	0.172 0.508 0.051
Nodal stage (N1 versus N0)	5.820	1.512	22.406	0.010	3.892	1.065	14.227	0.040
Orbital invasion (yes versus no)	4.131	1.476	11.567	0.007	3.175	1.267	7.960	0.014
Intracranial extension (yes versus no)	3.340	1.135	9.826	0.029	2.909	1.134	7.463	0.026
Surgery versus no surgery	0.173	0.059	0.512	0.002	0.293	0.117	0.730	0.008
RT versus no RT	0.220	0.067	0.729	0.013	0.153	0.053	0.440	0.001
Chemotherapy versus no chemotherapy	4.184	1.419	12.337	0.009	3.326	1.304	8.483	0.012

OS: Overall survival, DFS: Disease free survival
